# Chemogenetic silencing of GABAergic dorsal horn interneurons induces morphine-resistant spontaneous nocifensive behaviours

**DOI:** 10.1038/s41598-017-04972-3

**Published:** 2017-07-05

**Authors:** Keisuke Koga, Kensho Kanehisa, Yuta Kohro, Miho Shiratori-Hayashi, Hidetoshi Tozaki-Saitoh, Kazuhide Inoue, Hidemasa Furue, Makoto Tsuda

**Affiliations:** 1 Department of Life Innovation, Graduate School of Pharmaceutical Sciences, Kyushu University, Fukuoka, 812-8582 Japan; 2 Department of Molecular and System Pharmacology, Graduate School of Pharmaceutical Sciences, Kyushu University, Fukuoka, 812-8582 Japan; 3 Division of Neural Signaling, National Institute for Physiological Sciences, Okazaki, Aichi 444-8787 Japan; 4 Department of Neurophysiology, Hyogo College of Medicine, Nihinomiya, Hyogo, 663-8501 Japan

## Abstract

Inhibitory interneurons in the spinal dorsal horn (SDH) are crucial for processing somatosensory information originating in the periphery. However, the effects of the acute and selective inactivation of GABAergic SDH interneurons on pain processing are not fully understood. In this study, we used designer receptors exclusively activated by designer drugs (DREADD) technology and vesicular GABA transporter-Cre (*Vgat-Cre*) mice to selectively express a modified human muscarinic Gi protein-coupled receptor (hM4Di) in *Vgat-Cre* ^+^ GABAergic SDH interneurons in the fourth lumbar segment. We found that clozapine-N-oxide (CNO) treatment rapidly hyperpolarized these neurons and induced spontaneous nocifensive behaviours in these mice. In *Vgat-Cre* ^neg^ lamina II neurons, CNO produced facilitation of A fibre-mediated polysynaptic excitatory responses, an effect that required *N*-methyl-D-aspartate (NMDA) receptor activation. The CNO-induced nocifensive behaviours were also reduced by NMDA receptor antagonism. Moreover, these nocifensive behaviours were suppressed by pregabalin but resistant to morphine. Our findings indicate that *Vgat-Cre* ^+^ SDH interneurons play an important role in morphine-resistant nocifensive behaviours and suggest that this approach may provide a useful model for understanding the mechanisms of opioid-resistant pain signalling and for developing novel analgesics.

## Introduction

Somatosensory information from the periphery is conveyed to the spinal dorsal horn (SDH) via primary afferents^1^. A complex network consisting of a number of excitatory and inhibitory interneurons exists within the SDH, and it is the balance between the excitation and inhibition in these SDH neural circuits that is thought necessary for the proper processing of peripherally input somatosensory information and for producing normal sensation^2^. Indeed, pharmacological blockade of receptors for the inhibitory neurotransmitters γ-aminobutyric acid (GABA) and glycine in the spinal cord has been shown to produce pain hypersensitivity to innocuous mechanical stimulation, hyperalgesia, and spontaneous pain^2^. However, the exact role of the SDH inhibitory interneurons in somatosensory processing is not fully understood because GABA and glycine in the spinal cord are released not only from SDH interneurons but also from neurons descending from the brainstem, such as from the rostral ventromedial medulla (RVM)^3, 4^. Furthermore, although recent studies have reported exaggerated pain responses by ablating or silencing SDH inhibitory interneurons using diphtheria toxin or tetanus toxin, respectively^5, 6^, because of possible secondary nonspecific adverse effects resulting from the neuronal damage inflicted by these manipulations, it is important to determine the impact of SDH inhibitory interneurons on pain processing using an approach that allows acute and selective manipulation of the function of these neurons.

Therefore, in this study, we used designer receptors exclusively activated by designer drugs (DREADD) technology^7^ to acutely silence neurons^8^. For the selective manipulation of SDH inhibitory interneurons, we used the flip-excision (FLEX) system^9, 10^ in which an adeno-associated virus (AAV) encoding a modified human muscarinic Gi protein-coupled receptor (hM4Di) was microinjected using our established minimally invasive injection method into the SDH^11^ of *Vgat-Cre* mice^12^ containing genetically targeted inhibitory interneurons. Using this system, we examined the effects of silencing SDH inhibitory interneurons on somatosensory information processing at both the synaptic and behavioural levels.

## Results

### *Vgat-Cre*^+^ SDH interneuron-specific hM4Di expression

**Figure 1:**
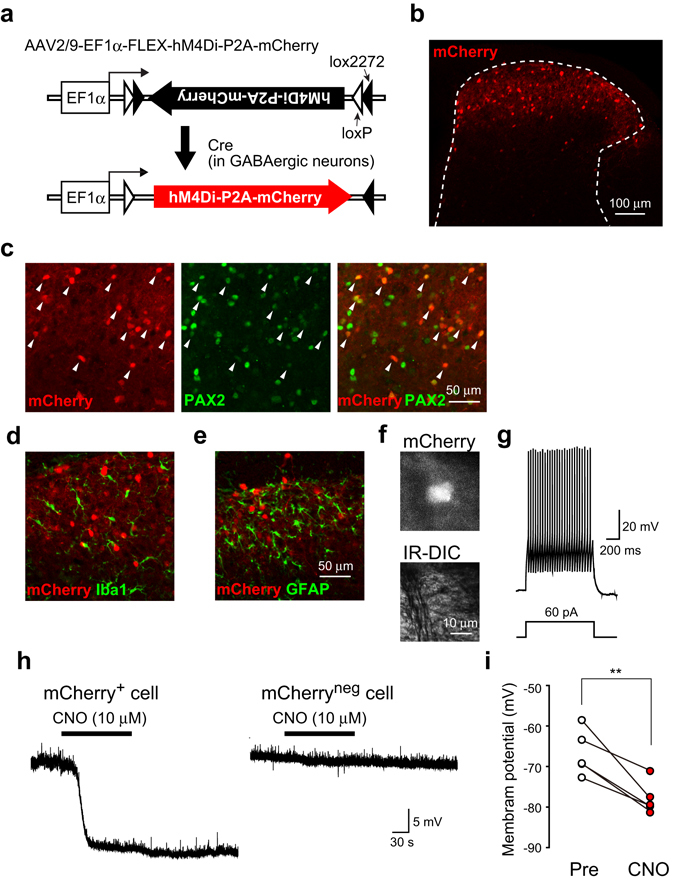
*Vgat-Cre* ^+^ SDH interneuron-specific hM4Di expression. (**a**) Schematic illustration of the FLEX switch system. AAV2/9 vector carrying EF1α and an inverted hM4Di-P2A-mCherry sequence flanked by loxP and lox2272 on both sides. Cre-dependent genetic inversion switch of the hM4Di-P2A-mCherry sequence occurs in Cre-expressing GABAergic SDH interneurons of *Vgat-Cre* mice. (**b**) mCherry expression in the SDH of mice 4 weeks after intra-SDH injection of AAV2/9-FLEX-hM4Di-P2A-mCherry. (**c–e**) Immunohistochemical identification of mCherry-expressing cells using cell-type specific markers (green; (**c)** PAX2; (**d)** Iba1; (**e**) GFAP). Arrowheads indicated mCherry^+^ cells with PAX2 immunofluorescence. (**f**) Photomicrographs of mCherry^+^ SDH neurons (upper, epifluorescence; lower, Nomarski optics) in spinal cord slices taken from *Vgat-Cre*;AAV-hM4Di^FLEX^ mice. (**g**) Representative firing pattern of an mCherry^+^ SDH neuron in a spinal cord slice. (**h**) Representative traces of the effect of CNO (10 μM) on mCherry^+^ (left) and mCherry^neg^ (right) SDH neurons. (**i**) Quantification of membrane potentials in mCherry^+^ SDH neurons before (Pre) and after application of CNO (n = 5, **P < 0.01).

To selectively express hM4Di in SDH inhibitory interneurons, we unilaterally microinjected an hM4Di-encoded AAV (AAV2/9-EF1α-FLEX-hM4Di-P2A-mCherry) into the SDH of *Vgat-Ires-Cre* mice (termed *Vgat-Cre*;AAV-hM4Di^FLEX^) at the fourth lumbar segment, which mainly receives somatosensory inputs from the hindpaw. In *Vgat-Cre* ^+^ cells (GABAergic inhibitory neurons), a Cre-dependent genetic inversion switch occurs (Fig. 1a). Four weeks after the injection, mCherry fluorescence was detected exclusively in the ipsilateral SDH, predominantly in laminae I–III (Fig. 1b). Double immunostaining showed that 97.6 ± 1.1% of mCherry^+^ (*Vgat-Cre* ^+^) cells (total tested cells: 521, 4 slices, 4 mice) were immunolabelled with paired box 2 (PAX2) (Fig. 1c), a marker for inhibitory interneurons^13^. mCherry fluorescence in the SDH was observed in 25.1 ± 3.3% of total PAX2^+^ cells tested. In these counting, we included cells with high and low immunofluorescence of PAX2 as PAX2^+^ cells based on our preliminary data showing that there were no PAX2^+^ cells in sections taken from *Vgat-Cre*;AAV-hM4Di^FLEX^ mice when the sections were immunostained by the secondary antibody (Alexa 488) in the absence of PAX2 primary antibody (Supplementary Fig. 1). In addition, mCherry^+^ cells were not immunolabelled with ionized calcium-binding adapter molecule-1 (Iba1) (Fig. 1d) or with glial fibrillary acidic protein (GFAP) (Fig. 1e), markers of microglia and astrocytes, respectively. To determine whether *Vgat-Cre* ^+^ SDH interneurons were inhibited by activation of hM4Di, we performed whole-cell patch-clamp recordings. In spinal cord slices taken from *Vgat-Cre*;AAV-hM4Di^FLEX^ mice, mCherry fluorescence was clearly visible in *Vgat-Cre* ^+^ SDH interneurons (Fig. 1f). Under current-clamp mode, all mCherry^+^ SDH neurons tested (n = 5) exhibited a tonic firing pattern after a depolarizing current injection (Fig. 1g), which has been reported in SDH inhibitory interneurons^2^. Bath application of clozapine N-oxide (CNO), a synthetic compound activating hM4Di, rapidly hyperpolarized the resting membrane potentials of *Vgat-Cre* ^+^ SDH neurons (Fig. 1h,i), but not of mCherry-negative neurons (*Vgat-Cre* ^neg^ neurons; Fig. 1h). These results indicate that under our experimental conditions, CNO acutely and specifically silences the activity of *Vgat-Cre* ^+^ GABAergic SDH interneurons.

### **Silencing*Vgat-Cre***^**+**^**SDH interneurons elicits spontaneous nocifensive behaviours**

**Figure 2:**
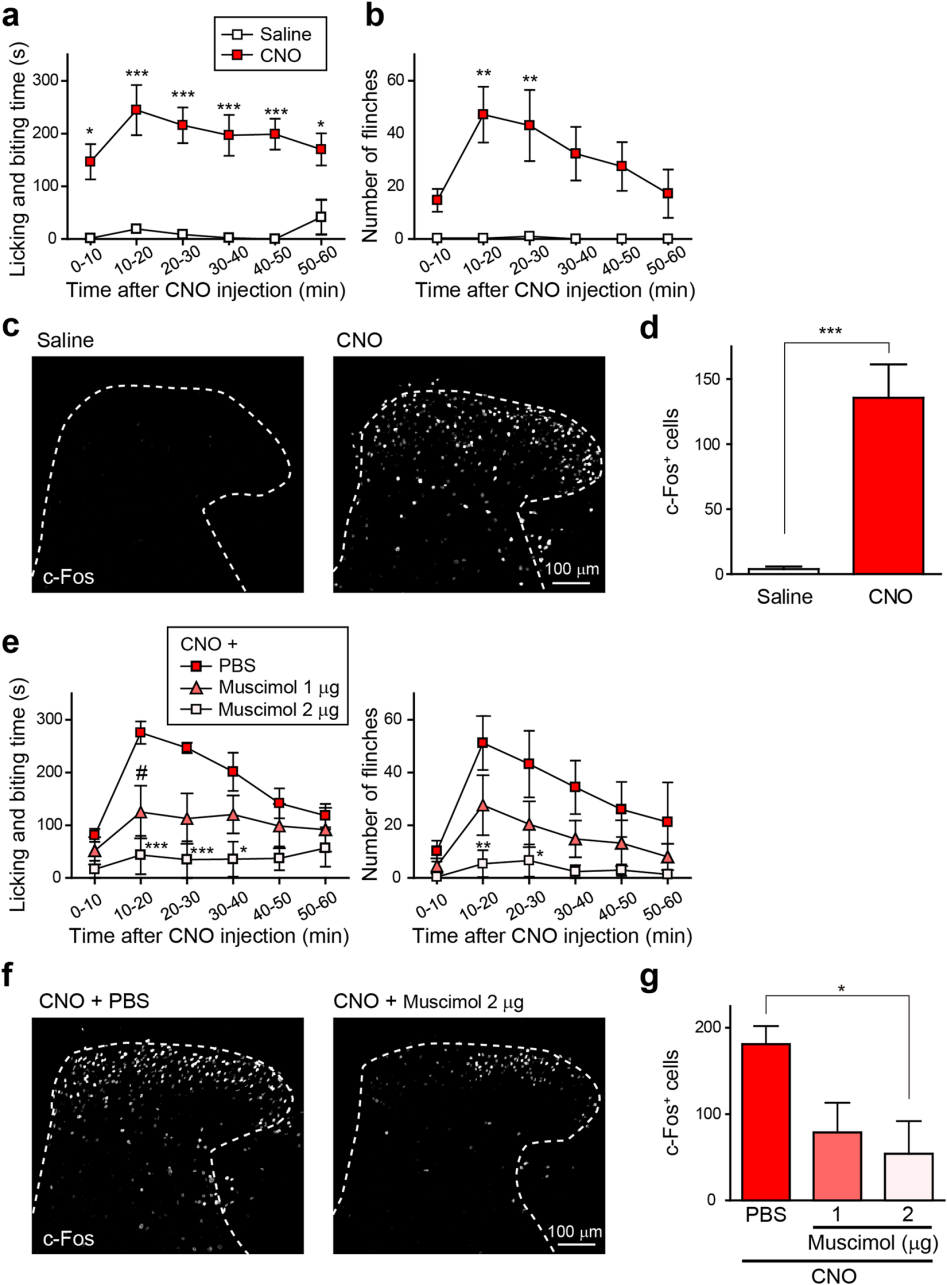
Silencing *Vgat-Cre* ^+^ SDH interneurons elicits spontaneous nocifensive behaviours. (**a,b**) Spontaneous nocifensive behaviours, licking and biting (**a**) and flinching (**b**) after i.p. injection of saline or CNO (10 mg kg^−1^) in *Vgat-Cre*;AAV-hM4Di^FLEX^ mice. These behaviours were assessed for 1 hr after CNO injection (n = 4–6; *P < 0.05, **P < 0.01, ***P < 0.001 vs. saline). (**c**) c-Fos immunoreactivity in the SDH of *Vgat-Cre*;AAV-hM4Di^FLEX^ mice 90 min after administration of saline or CNO (10 mg kg^−1^, i.p.). (**d**) Quantification of c-Fos^+^ cells in the SDH (n = 5, ***P < 0.001). (**e**) Effects of intrathecal pretreatment with muscimol, a GABA_A_ receptor agonist, on the CNO-induced nocifensive behaviours (left, licking and biting; right, flinching); n = 5; *P < 0.05, **P < 0.01, ***P < 0.001, ^#^ *P* < 0.05 vs. CNO + PBS. (**f,g**) Effect of intrathecal pretreatment with muscimol (1 and 2 μg) on the CNO-induced increase in c-Fos^+^ cells. c-Fos immunoreactivity in the SDH (**f**) and quantification ((**g)** n = 5, *P < 0.05). Data are means ± SEM.

To examine the effects of chemogenetically silencing *Vgat-Cre* ^+^ SDH interneurons on behaviour, we intraperitoneally administered CNO to *Vgat-Cre*;AAV-hM4Di^FLEX^ mice. We found that CNO-injected mice displayed robust spontaneous licking and biting of their hindpaw ipsilateral to the hM4Di expression (Fig. 2a). The CNO-induced licking and biting peaked 10–20 min post-injection and persisted until at least the final tested time point (Fig. 2a and Supplementary Video 1). CNO also induced flinching behaviour with a time course similar to that for licking and biting (Fig. 2b). We next investigated neuronal activity by examining c-Fos immunostaining. After CNO administration, we observed many c-Fos^+^ cells in the SDH (Fig. 2c), and the number of c-Fos^+^ cells was significantly increased (Fig. 2d). Given that the CNO-induced spontaneous nocifensive behaviours resulted from the silencing of GABAergic interneurons, we reasoned that stimulating GABA_A_ receptors would rescue these phenotypes. We found that intrathecal pretreatment with muscimol, a GABA_A_ receptor agonist, effectively prevented the CNO-induced nocifensive behaviours (Fig. 2e) and c-Fos expression (Fig. 2f,g). Therefore, these findings indicate that chemogenetic removal of the inhibitory tone of *Vgat-Cre* ^+^ SDH interneurons elicits nocifensive behaviours and SDH neuronal excitability.

### CNO-induced facilitation of A fibre-mediated synaptic responses in lamina II neurons

**Figure 3:**
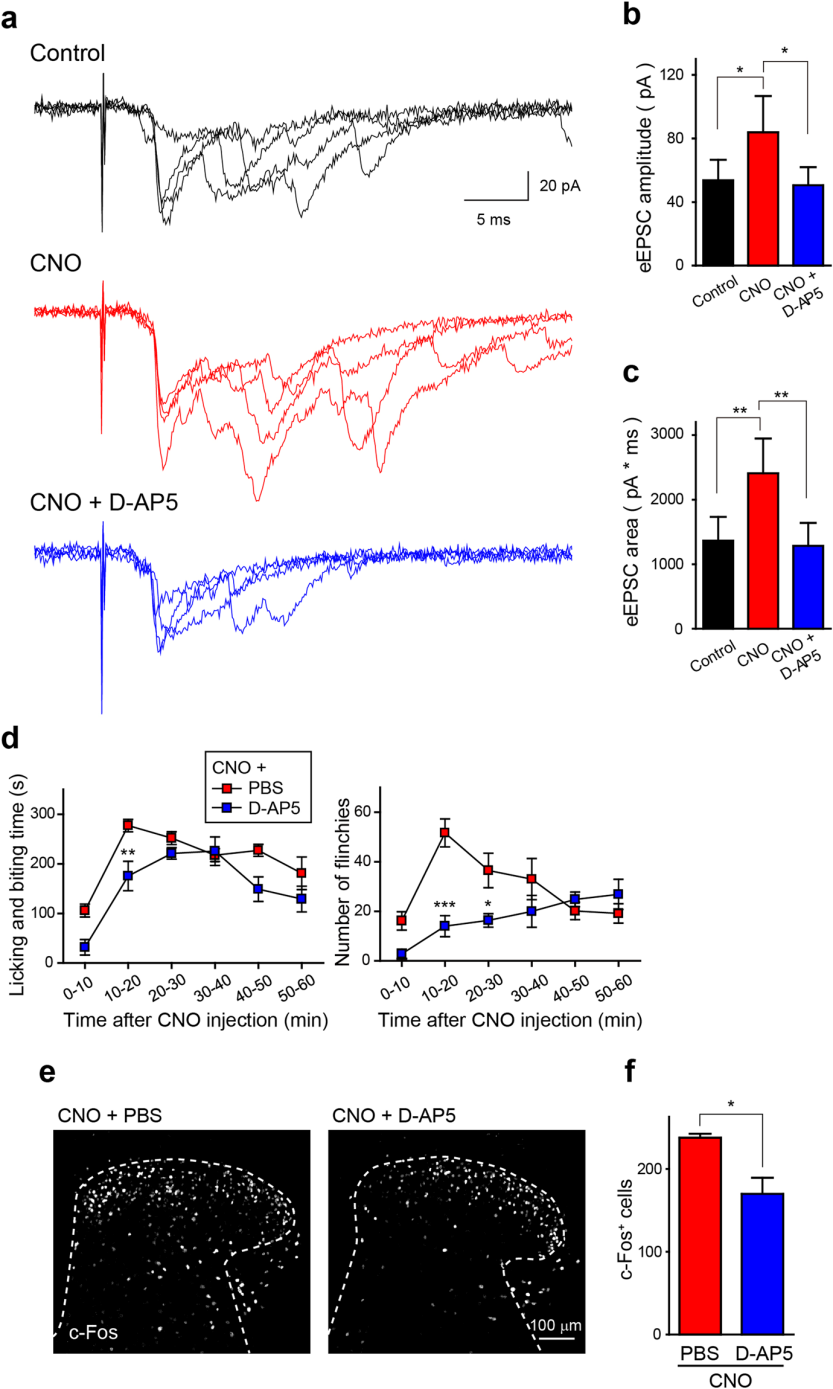
CNO-induced facilitation of A fibre-mediated polysynaptic responses in lamina II neurons of *Vgat-Cre*;AAV-hM4Di^FLEX^ mice. (**a**) Representative polysynaptic excitatory postsynaptic currents (EPSCs) evoked by A fibres stimulation (100 μA for 100 μs) in *Vgat-Cre* ^neg^ lamina II neurons before (top) and after bath application of CNO (10 μM; middle) and CNO with D-AP5 (50 μM; bottom). (**b**) Quantification of the amplitude of polysynaptic A fibre-evoked EPSCs (eEPSCs) (n = 6; *P < 0.05). (**c)** Quantification of the area of eEPSCs. Area was calculated by measuring the integrated area of eEPSCs (0–200 ms) (n = 6; **P < 0.01). (**d,e**) Effect of intrathecal pretreatment with D-AP5 (5 μg/5 μL) or PBS on the CNO-induced nocifensive behaviours (left, licking and biting; right, flinching) ((**d)** n = 6, *P < 0.05, **P < 0.01, ***P < 0.001 vs. CNO + PBS) and on the CNO-induced increase in c-Fos^+^ cells in the SDH. c-Fos immunoreactivity in the SDH (**e**) and quantification ((**f)** n = 6, *P < 0.05). Data are means ± SEM.

Pharmacological blockade of spinal inhibitory neurotransmission facilitates excitation of lamina II neurons evoked by A fibre stimulation^14, 15^. We performed whole-cell patch-clamp recordings in *Vgat-Cre* ^neg^ lamina II neurons using parasagittal spinal cord slices with attached dorsal roots obtained from *Vgat-Cre*;AAV-hM4Di^FLEX^ mice (Supplementary Fig. 2) and examined the effects of CNO on A fibre-mediated excitatory postsynaptic currents (EPSCs). In recorded neurons, polysynaptic EPSCs evoked by electrical stimulation of A fibres (100 μA for 100 μs)^16, 17^ was facilitated by bath application of CNO (Fig. 3a). In neurons that received A fibre inputs mono- and poly-synaptically, the facilitating effect of CNO was also mainly observed in polysynaptic EPSCs (Supplementary Fig. 3). CNO significantly increased the polysynaptic EPSC amplitude (Fig. 3b) and integrated area of the A fibre-evoked EPSCs (0–200 ms after A fibre stimulation) (Fig. 3c). The CNO-induced facilitation was eliminated by application of D-(-)-2-amino-5-phosphonopentanoic acid (D-AP5), an *N*-methyl-D-aspartate (NMDA) receptor antagonist (Fig. 3a–c and Supplementary Fig. 3), suggesting that silencing *Vgat-Cre* ^+^ SDH interneurons leads to facilitation of A fibre-evoked excitation of lamina II neurons through the activation of NMDA receptors. We also found that intrathecal administration of D-AP5 (5 μg/5 μL) significantly attenuated the CNO-induced licking, biting, and flinching behaviours (Fig. 3d) as well as the increase in c-Fos^+^ cells in the SDH (Fig. 3e,f). These results suggest that the activation of NMDA receptors is involved in the facilitation of A fibre-mediated excitatory postsynaptic responses in lamina II neurons and of nocifensive behaviours evoked following the silencing of *Vgat-Cre* ^+^ SDH inhibitory interneurons.

### CNO-induced nocifensive behaviours are resistant to morphine

**Figure 4:**
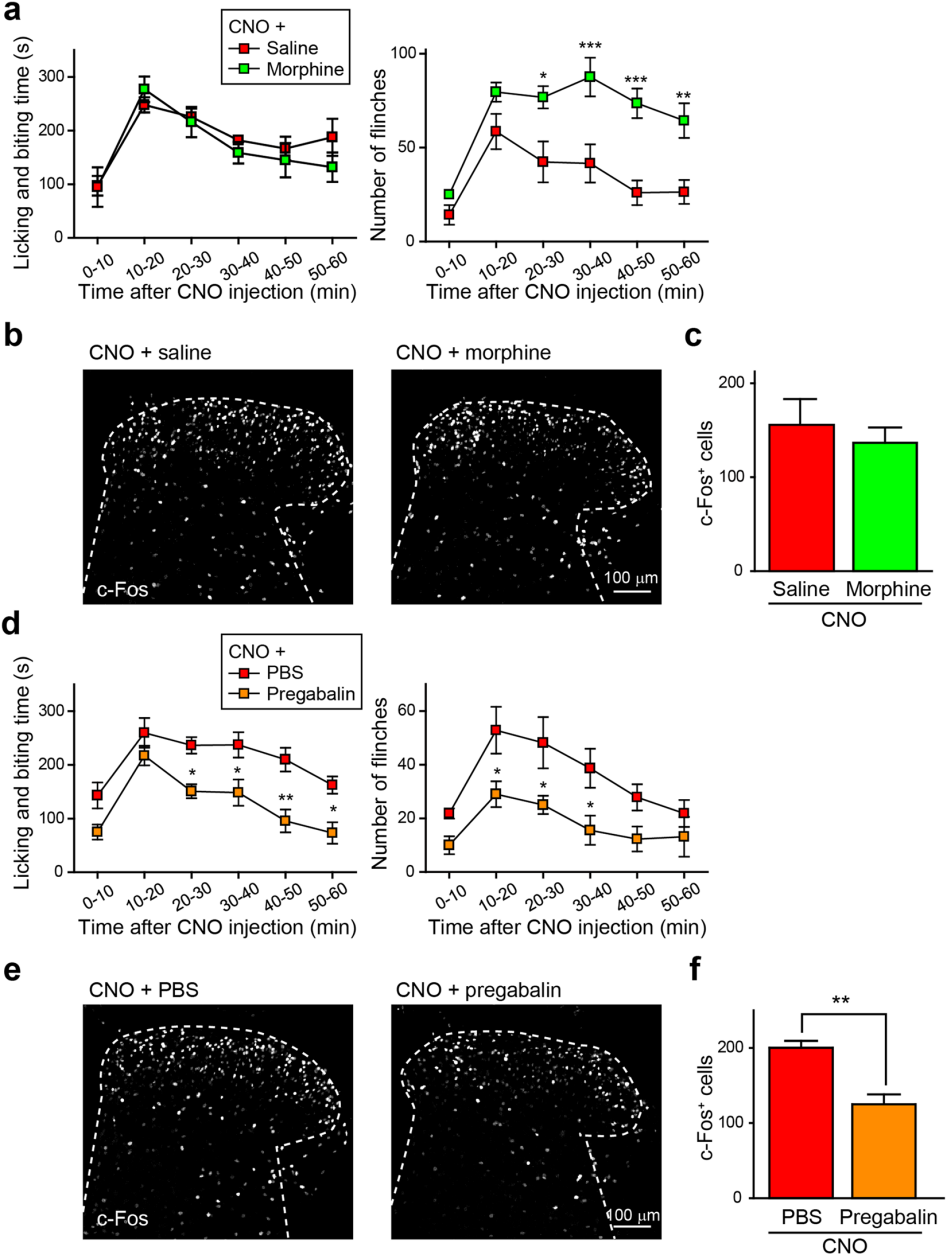
Effect of morphine and pregabalin on CNO-induced spontaneous nocifensive behaviours. (**a,b**) Effects of morphine (3 mg kg^−1^, s.c.) on CNO-induced nocifensive behaviours (left, licking and biting; right, flinching) ((**a)** n = 5; *P < 0.05, **P < 0.01, ***P < 0.001 vs. CNO + saline) and on c-Fos^+^ cells in the SDH. c-Fos immunoreactivity in the SDH (**b**) and quantification ((**c)** n = 5). (**d–f**) Effects of intrathecal administration of pregabalin (10 μg/5 μL) on CNO-induced nocifensive behaviours (left, licking and biting; right, flinching) (**(d)** n = 6–7; *P < 0.05, **P < 0.01 vs. CNO + PBS) and on c-Fos^+^ cells in the SDH. c-Fos immunoreactivity in the SDH (**e**) and quantification ((**f)** n = 7; **P < 0.01). Data are means ± SEM.

Finally, we examined the effects of morphine and pregabalin on the CNO-induced nocifensive behaviours in *Vgat-Cre*;AAV-hM4Di^FLEX^ mice. Subcutaneous administration of morphine (3 mg kg^−1^) had no effect on the CNO-induced licking and biting behaviours and even enhanced the CNO-generated flinching (Fig. 4a). Moreover, the increase in c-Fos^+^ cells in the SDH observed after CNO administration was not decreased by morphine (Fig. 4b,c). By contrast, intrathecal administration of pregabalin (10 μg/5 μL) significantly suppressed both the CNO-induced nocifensive behaviours (Fig. 4d) and c-Fos expression (Fig. 4e,f). Thus, nocifensive behaviours produced by silencing *Vgat-Cre* ^+^ SDH inhibitory interneurons are attenuated by pregabalin but resistant to morphine.

## Discussion

GABAergic inhibitory signals in the SDH are crucial for regulating somatosensory information processing. Previous pharmacological studies have shown that intrathecal administration of the GABA_A_ receptor antagonist bicuculline to normal animals produces pain hypersensitivity to innocuous mechanical stimulation, hyperalgesia, and spontaneous pain^2^. GABA is released not only from SDH interneurons but also from neurons descending from the brainstem, such as from the RVM^3, 4^. In the present study, we used chemogenetic manipulation of a selective population of GABAergic SDH interneurons to first show the highly selective expression of hM4Di in *Vgat-Cre* ^+^ SDH inhibitory interneurons and their silencing by CNO and then to demonstrate for the first time that the rapid and selective silencing of these interneurons caused robust spontaneous nocifensive behaviours. Intrathecal injection of the GABA_A_ agonist muscimol rescued both the behavioural phenotype and the c-Fos induction caused by CNO. The microinjection method used here has been reported to express genes of interest in SDH neurons but not in dorsal root ganglion (DRG) neurons^18^, strengthening our contention that *Vgat-Cre* ^+^ SDH interneurons play an important role in controlling somatosensory information processing under normal conditions.

In our electrophysiological analysis of *Vgat-Cre* ^neg^ lamina II neurons, the acute inhibition of *Vgat-Cre* ^+^ SDH interneurons by CNO facilitated A fibre-evoked polysynaptic responses. It is conceivable that *Vgat-Cre* ^+^ SDH interneurons may predominantly regulate A fibre-mediated excitatory polysynaptic transmission. It was previously reported that antagonism of spinal GABA_A_ receptors facilitates A fibre-mediated (and to a much lesser extent C fibre-mediated) excitatory polysynaptic responses^14^, which supports the present findings. The CNO-induced facilitation of A fibre-evoked synaptic responses might involve the activation of NMDA receptors because the NMDA receptor antagonist D-AP5 prevented the CNO-induced synaptic facilitation. Furthermore, this antagonist also reduced both the nocifensive behaviours and c-Fos induction. Baba *et al*.^14^ have reported that bicuculline does not change the frequency of miniature EPSCs, suggesting that bicuculline may not facilitate the release of excitatory transmitters, including glutamate. Thus, removing the inhibitory tone derived from *Vgat-Cre* ^+^ SDH interneurons may result in eliminating the Mg^2+^ block of NMDA receptors in *Vgat-Cre* ^neg^ lamina II neurons, which would facilitate the excitatory synaptic inputs from A fibres.

We also found that the CNO-induced nocifensive behaviours were resistant to morphine. In fact, morphine did not suppress the CNO-induced licking and biting and even enhanced flinching. The reason for the enhancement remains unclear, but in these mice, flinching appears to be followed by movement (see Supplementary Video 2). The time spent in exploratory movement was markedly increased by morphine, for example, 40–50 min after CNO injection (CNO + saline, n = 5, 21.3 ± 5.7 s; CNO + morphine, n = 5, 154 ± 22.6 s; *P* < 0.01, unpaired *t*-test). It is thus plausible that the morphine-induced increase in flinching was caused by augmentation of the mechanical signalling inputs (presumably via A fibres) associated with movement. Ineffectiveness of morphine has been reported in animal models of neuropathic pain, especially in pain hypersensitivity to light mechanical stimulation of the peripheral skin^19, 20^. Consistent with this, dysfunction of inhibitory neurotransmission has been shown after nerve injury in several studies^15, 21, 22^. Morphine exerts its analgesic effects via μ-opioid receptors (MORs), which are expressed in pain neural circuits. A recent study has shown that genetic deletion of MORs in primary afferent nociceptors has no effect on antinociception by morphine systemically administered^23^. Morphine also activates RVM serotonergic cells and causes serotonergic input to the SDH. Serotonin activates spinal inhibitory interneurons^24, 25^, and morphine administered intracerebroventricularly causes GABA release in the spinal cord via activation of 5HT_3_ receptors, which are involved in the antinociceptive effect of morphine^26^. Therefore, it is possible that the chemogenetic silencing of *Vgat-Cre* ^+^ SDH interneurons may prevent the effect of morphine in these neurons and may explain the behavioural resistance to morphine. However, pregabalin, which suppressed CNO-induced nocifensive behaviours, reportedly acts on the voltage-dependent Ca^2+^ channel subunit α2δ^27^ to inhibit spinal neurotransmission from primary afferents^28, 29^, which may explain the pregabalin sensitivity to the CNO-induced pain. Thus, *Vgat-Cre* ^+^ SDH interneurons appear to be an important component in SDH neuronal circuits that are insensitive to morphine. Therefore, chemogenetically evoked pain behaviours in *Vgat-Cre* mice may be useful for elucidating the mechanisms underlying the opioid ineffectiveness observed in neuropathic pain conditions and also for discovering potent analgesics for treating chronic pain.

In conclusion, we demonstrated that the rapid and selective silencing of GABAergic SDH interneurons using DREADD technology induced morphine-resistant spontaneous nocifensive behaviours and produced facilitation of A fibre-mediated excitatory polysynaptic responses in non-GABAergic lamina II neurons. These results suggest that this approach may provide a useful model for understanding the mechanisms of opioid-resistant pain signalling and for developing novel analgesics.

## Methods

### Animals

*Vgat-Ires-Cre* mice (Stock No: 016962, The Jackson Laboratory)^12^ and C57BL/6J mice (Stock No: 000664) were used. Homozygous *Vgat-Ires-Cre* mice were crossed once with C57BL/6J mice to generate heterozygous *Vgat-Ires-Cre* mice. All mice used were male heterozygous *Vgat-Ires-Cre* mice, 8–12 weeks of age at the start of each experiment and were housed at 22 ± 1 °C with a 12-h light-dark cycle. All animals were fed food and water *ad libitum*. All animal experiments were conducted according to relevant national and international guidelines contained in the ‘Act on Welfare and Management of Animals’ (Ministry of Environment of Japan) and ‘Regulation of Laboratory Animals’ (Kyushu University) and under the protocols approved by the Institutional Animal Care and Use committee review panels at Kyushu University.

### Recombinant adeno-associated virus (rAAV) vector production

To produce rAAV vector for Cre-dependent gene transduction, a vector containing the EF1α promoter was generated from pAAV-CA-FLEX (Addgene plasmid #38042)^30^, by substituting the CA promoter with EF1α. We amplified hM4Di (Addgene plasmid #45548)^31^ and mCherry and purchased 2A sequence. Then, we cloned them into the above pAAV-EF1α-FLEX to generate pAAV-EF1α-hM4Di-2A-mCherry. The rAAV vector was produced from human embryonic kidney 293 (HEK293) cells with triple transfection [pZac, cis plasmid; pAAV2/9, trans plasmid; pAd DeltaF6, adenoviral helper plasmid (All plasmids were purchased from the University of Pennsylvania Gene Therapy Program Vector Core)] and purified by two cesium chloride density gradient purification steps. The vector was dialyzed against phosphate-buffered saline (PBS) containing 0.001% (v/v) Pluronic-F68 using Amicon Ultra 100K filter units (Millipore). The Genome titre of rAAV was determined by PicoGreen fluorometric reagent (Molecular Probes) following denaturation of the AAV particle. Vectors were stored in aliquots at −80 °C until use^11^.

### Intra-SDH injection of rAAV vector

Mice were deeply anaesthetized by subcutaneous (s.c.) injection of ketamine (100 mg kg^−1^) and xylazine (10 mg kg^−1^). The skin was incised at Th12–L3, and custom-made clamps were attached to the rostral and caudal sites of the vertebral column. Paraspinal muscles around the left side of the interspace between Th13 and L1 vertebrae were removed, and the dura mater and the arachnoid membrane were carefully incised using the tip of a 30G needle to make a small window. Then, we inserted the microcapillary backfilled with rAAV solution into the SDH (500 μm lateral from the midline and 150 μm in depth from the surface of the dorsal root entry zone) and microinjected 500 nL rAAV solution (1 × 10^12^ GC mL^−1^) using Ultra Micro Pump (MICRO4, Word Precision Instruments). After microinjection, the inserted microcapillary was removed from the SDH, the skin was sutured with 3-0 silk, and mice were kept on a heating pad until recovery.

### Behavioural studies and c-Fos analysis

For behavioural studies, mice 4–5 weeks after viral injection were used. Mice were placed in clear plastic containers (11 cm in diameter, 18 cm high) for observation and allowed to acclimate for 30 min prior to behavioural assessments. All assays were recorded, and subsequently scored time of licking and biting of hind paw on one side, or number of paw flinching (rapid flicking of the limb) on one side after CNO (10 mg kg^−1^ in saline, Enzo Life Sciences) or saline intraperitoneal injection for 1 hr. To analyse the effect of analgesic drugs, we intrathecally injected muscimol (1 or 2 μg/5 μL in PBS, Abcam), D-AP5 (5 μg/5 μL in PBS, Abcam), pregabalin (10 μg/5 μL in PBS, Sigma), or PBS (for control) under isoflurane (2%) anaesthesia^32^ 10 min before CNO (10 mg kg^−1^) injection. To analyse the effect of morphine, we subcutaneously injected morphine (3 mg kg^−1^) or saline (for control) 10 min before CNO (10 mg kg^−1^) injection. To investigate the expression of c-Fos after CNO injection, we quickly perfused the mice 90 min after CNO injection as described below.

### Immunohistochemistry

Mice were deeply anaesthetized by i.p. injection of pentobarbital and perfused transcardially with PBS, followed by ice-cold 4% paraformaldehyde/PBS. The L3–4 (transverse) segments of the spinal cord were removed, postfixed in the same fixative for 3 h at 4 °C, and placed in 30% sucrose solution for 24 h at 4 °C. Transverse L4 spinal cord sections (30 μm) were incubated in blocking solution (3% normal goat serum or normal donkey serum) for 2 h at room temperature and then incubated for 48 h at 4 °C with primary antibodies: polyclonal rabbit anti-PAX2 (1:1000, Invitrogen), monoclonal rat anti-GFAP (1:2000, Invitrogen), monoclonal mouse anti-NeuN (1:2000, Millipore), polyclonal rabbit anti-Iba1 (1:5000, Wako), and polyclonal rabbit anti-c-Fos (1:5000, Santa Cruz). Following incubation, tissue sections were washed and incubated for 3 h at room temperature in secondary antibody solution (Alexa Fluor 488 and/or 405, 1:1000, Molecular Probes). The tissue sections were washed, slide-mounted and subsequently coverslipped with Vectashield hardmount (Vector Laboratories). Three to five sections from the L4 spinal cord segments of each mouse were randomly selected and analysed using a LSM 700 Imaging System (Carl Zeiss).

### Spinal cord slice

*Vgat-Cre*;AAV-hM4Di^FLEX^ mice were deeply anesthetized with urethane (1.2–1.5 g kg^−1^, i.p.) and thoracolumbar laminectomy was performed. The lumbosacral spinal cord was removed and placed into an ice-cold high sucrose artificial cerebrospinal fluid (250 mM sucrose, 2.5 mM KCl, 2 mM CaCl_2_, 2 mM MgCl_2_, 1.2 mM NaH_2_PO_4_, 25 mM NaHCO_3_ and 11 mM glucose). After cutting all of the ventral and dorsal roots except for the L3 or L4 dorsal root on one side. Parasagittal spinal cord slices (250–300 μm thick) with L3 or L4 dorsal root attached were made using a vibrating microtome (VT1200, Leica) and then the slices kept in oxygenated artificial cerebrospinal fluid (aCSF) solution (125 mM NaCl, 2.5 mM KCl, 2 mM CaCl_2_, 1 mM MgCl_2_, 1.25 mM NaH_2_PO_4_, 26 mM NaHCO_3_ and 20 mM glucose) at room temperature (22–25 ^o^C) for at least 30 min.

### Whole-cell patch-clamp recording

The spinal cord slice was then put into a recording chamber where it was continuously superfused with aCSF solution at a flow rate of 4–7 ml min^−1^. Lamina II was identified as a translucent band across the dorsal horn and whole-cell recordings were made from the neurons in the lamina II. The patch pipettes were filled with an internal solution (125 mM K-gluconate, 10 mM KCl, 0.5 mM EGTA, 10 mM HEPES, 4 mM ATP-Mg, 0.3 mM NaGTP, 10 mM phosphocreatine, 0.4% Neurobiotin, pH 7.28 adjusted with KOH). The tip resistance of the patch pipettes was 4–6 MΩ. Recordings were made using Axopatch 700B amplifier and pCLAMP 10.4 acquisition software (Molecular Devices). The data were digitized with an analog-to-digital converter (Digidata 1550, Molecular Devices), stored on a personal computer using a data acquisition program (Clampex version 10.4, Molecular Devices) and analysed using a software package (Clampfit version10.4, Molecular Devices). The membrane potentials were recorded in the current-clamp mode and the firing patterns of lamina II neurons were determined by passing 1.0 s depolarizing current pulses through the recording electrode at resting membrane potential. Excitatory postsynaptic currents (EPSCs) were recorded in the voltage-clamp mode at a holding potential of −70 mV. The dorsal roots were stimulated with a suction electrode at A fibre strength (100 μA for 100 μs)^16, 17^ at 0.05 Hz for evoked EPSCs (eEPSC) recordings. The A fibre-evoked responses were considered monosynaptic if the latency remained constant when the root was stimulated at 20 Hz and there was no failure regardless of the constancy of the latency. In recorded *Vgat-Cre* ^neg^ lamina II neurons receiving A fibre-evoked polysynaptic inputs with or without monosynaptic (Supplementary Fig. 3 and Fig. 3a, respectively), the amplitude of polysynaptic EPSCs was calculated by averaging the peak amplitude of 5 evoked EPSC traces. The area of eEPSCs were calculated by measuring the integrated area of A fibre-evoked EPSCs (0–200 ms from stimulation artifact). All drugs were dissolved into the aCSF solution. CNO was bath-applied for 2 min in experiment related to Fig. 1h and 2 min before and during eEPSC recording in experiments related to Fig. 3a and Supplementary Fig. 2. Drugs used were CNO (10 μM, Enzo Life Sciences) and D-AP5 (50 μM, Abcam).

### Statistical analysis

All data are shown as the mean ± SEM. Statistical significance of differences was determined using paired t-test (Fig. 1i), unpaired t-test (Figs 2d, 3f, and 4c,f), two-way repeated measures ANOVA with *post hoc* Bonferroni test (Figs 2a,b,e, 3d, and 4a,d), one-way repeated measures ANOVA with *post hoc* Tukey’s multiple comparisons test (Fig. 3b,c), one-way ANOVA with *post hoc* Dunnett’s multiple comparisons test (Fig. 2g) using GraphPad Prism 7 software. Differences were considered significant at *P* < 0.05.

## Electronic supplementary material

Supplementary Information

Supplementary Video 1

Supplementary Video 2
