# Supplementary material for: Chemogenetic silencing of GABAergic dorsal horn interneurons induces morphine-resistant spontaneous nocifensive behaviours
Source: Sci Rep. 2017 Jul 5;7:4739. doi: 10.1038/s41598-017-04972-3 (PMC5498492; doi:10.1038/s41598-017-04972-3)
Supplement: Supplementary file 1 — Supplementary Information [file 41598_2017_4972_MOESM1_ESM.pdf]

Supplementary Information

Chemogenetic silencing of GABAergic dorsal horn interneurons induces morphine-resistant spontaneous nocifensive behaviours

Keisuke Koga, Kensho Kanehisa, Yuta Kohro, Miho Shiratori-Hayashi, Hidetoshi Tozaki-Saitoh, Kazuhide Inoue, Hidemasa Furue, Makoto Tsuda

Supplementary Figure 1

mCherry and PAX2 immunofluorescence in the SDH of *Vgat-Cre*;AAV-hM4Di^{FLEX} mice

mCherry (red) and immunofluorescence of PAX2 (green) and NeuN (blue) in spinal sections of *Vgat-Cre*;AAV-hM4Di^{FLEX} mice in the presence (upper panels) or absence (lower panels) of PAX2 primary antibody. PAX2 immunofluorescence with high and low intensity were indicated by arrow and arrowhead, respectively in upper panels.

Supplementary Figure 2

Whole-cell patch-clamp recording from *Vgat-Cre*^{neg} lamina II neurons

(**a**) Schematic illustration of whole-cell patch-clamp recording. A suction electrode was used to stimulate dorsal root to evoke synaptic responses in recording neurons. (**b**) Representative morphology of *Vgat-Cre*^{neg} lamina II neurons and the bottom images showed separated images of top merge image in white dotted open square (green, neurobiotin; red, mCherry fluorescence).

Supplementary Figure 3

CNO-induced facilitation of polysynaptic EPSCs in *Vgat-Cre*$^{\mathbf{neg}}$ lamina II neurons receiving mono- and poly-synaptic A fibre inputs

Representative mono- and polysynaptic excitatory postsynaptic currents (EPSCs) evoked by A fibre stimulation (100 μA for 100 μs) in *Vgat-Cre*^{neg} lamina II neurons before (top) and after bath application of CNO (10 μM; middle) and CNO with D-AP5 (50 μM; bottom).

Supplementary Video 1

Nocifensive behaviours in *Vgat-Cre*;hM4Di$^{\mathbf{FLEX}}$ mouse with saline or CNO (during 10–20 min post-injection)

Note: saline-injected *Vgat-Cre*;hM4Di^{FLEX} mouse showed normal behaviours, but CNO-injected *Vgat-Cre*;hM4Di^{FLEX} mouse displayed robust spontaneous licking, biting and flinching of the hindpaw ipsilateral to the hM4Di expression (left side).

Supplementary Video 2

CNO-induced nocifensive behaviours in *Vgat-Cre*;hM4Di$^{\mathbf{FLEX}}$ mouse pretreated with saline or morphine (during 40–50 min post-CNO injection)

Note: the CNO-induced flinching behaviour was increased in morphine-pretreated *Vgat-Cre*;hM4Di^{FLEX} mouse.

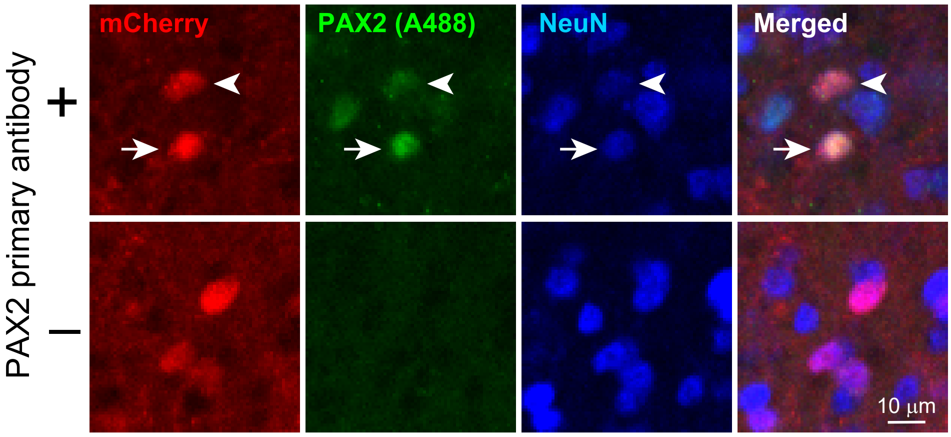

PAX2 primary antibody
+
–
mCherry
PAX2 (A488)
NeuN
Merged
10 µm

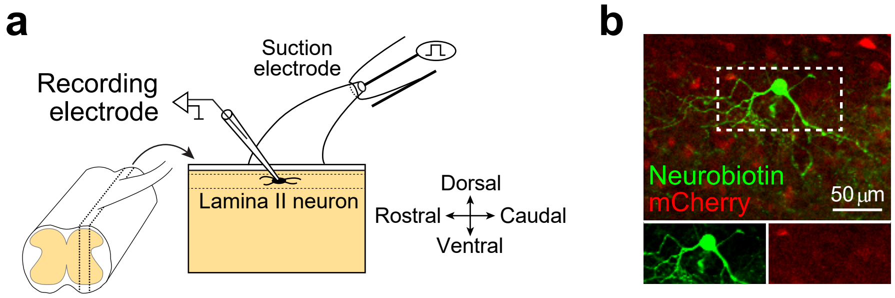
a
Suction electrode
Recording electrode
Lamina II neuron
Dorsal
Rostral
Caudal
Ventral
b
Neurobiotin
mCherry
50 μm

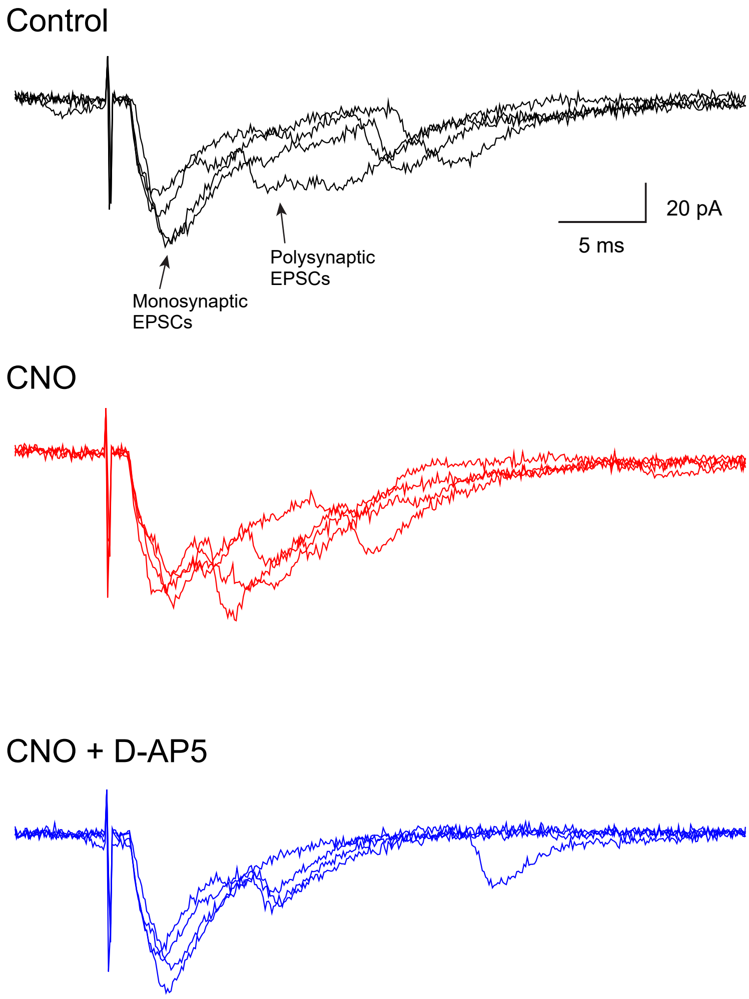
Control
20 pA
5 ms
Polysynaptic EPSCs
Monosynaptic EPSCs
CNO
CNO + D-AP5
